# Evaluation of 17-mm St. Jude Medical Regent prosthetic aortic heart valves by rest and dobutamine stress echocardiography

**DOI:** 10.1186/1749-8090-1-27

**Published:** 2006-09-19

**Authors:** Giovanni Minardi, Carla Manzara, Vittorio Creazzo, Daniele Maselli, Giovanni Casali, Giovanni Pulignano, Francesco Musumeci

**Affiliations:** 1 Department of Cardiology and Cardiovascular Surgery, Azienda Ospedaliera, S.Camillo-Forlanini, Rome, Italy

## Abstract

**Background:**

The prosthesis used for aortic valve replacement in patients with small aortic root can be too small in relation to body size, thus showing high transvalvular gradients at rest and/or under stress conditions. This study was carried out to evaluate rest and Dobutamine stress echocardiography (DSE) hemodynamic response of 17-mm St. Jude Medical Regent (SJMR-17 mm) in relatively aged patients at mean 24 months follow-up.

**Methods and results:**

The study population consisted of 19 patients (2 men, 17 women, mean age 69.2 ± 7.3 years). All patients underwent rest Doppler echocardiography before and after surgery and basal and DSE at follow up (infused at rate of 5 micrg/Kg/min and increased by 5 microg/Kg/min at 5 min intervals up to 40 microg/Kg/min). The following parameters were evaluated at rest and/or under DSE: heart rate (HR), ejection fraction (EF), cardiac output (CO), peak and mean velocity and pressure gradients (MxV, MnV, MxPG, MnPG), effective orifice area (EOA), indexed EOA (EOAi), left ventricular mass (LVM), indexed LVM (LVMi), Velocity Time Integral at left ventricular outflow tract (VTI LVOT) and transvalvular (Aortic VTI), Doppler velocity index (DVI). At rest MxPG and MnPG were 29.2 ± 7.1 and 16.6 ± 5.8mmHg, respectively; EOA and EOAi resulted 1.14 ± 0.3 cm^2^ and 0.76 ± 0.2 cm^2^/m^2^; DVI was normal (0.50 ± 0.1). At follow-up LVM and LVMi decreased significantly from pre-operative value of 258 ± 43g and 157.4 ± 27.7g/m^2^ to 191 ± 23.8g and 114.5 ± 10.6g/m^2^, respectively. DSE increased significantly HR, CO, EF, MxGP (up to 83.4 ± 2 1.9mmHg), MnPG (up to 43.2 ± 12.7mmHg). EOA, EOAi, DVI increased insignificantly (from baseline up to 1.2 ± 0.4 cm^2^, 0.75 ± 0.3cm^2^/m^2^ and 0.48 ± 0.1 respectively). Two patients developed significant intraventricular gradients.

**Conclusion:**

These data show that SJMR 17-mm prostheses can be safely implanted in aortic position in relatively aged patients, offering a satisfactory hemodynamic performance at rest and under DSE, with full utilization of its available orifice, suggesting that a possible mild prosthesis-patient mismatch is not an issue of clinical relevance when this small prosthesis is used. Rest and Dobutamine stress echocardiography is a useful and effective means for evaluating prosthesis hemodynamics and for monitoring the expected LVH regression.

## Background

Patients who have received prosthetic heart valves are usually followed by clinical evaluation and basal echocardiographic examinations [1,2]. Patients who receive a small aortic valve prosthesis may remain asymptomatic following surgery and Doppler echocardiography may show normal or mild elevated transvalvular gradients at rest, even in patients with large body surface area (BSA) [1-5]. However, this may not be representative of a patient's daily activities. Evaluation of valve hemodynamic response during stress conditions may offer useful information, simulating preclinical valve "dysfunction" [2,6,7]. Information derived from exercise stress echocardiography is limited because of the difficulty in obtaining adequate Doppler signals either due to the respiratory-related artefacts or to the increased chest wall motion during or immediately after exercise [2,6,7]. Recently, dobutamine stress echocardiography (DSE) has been proposed as an alternative and equally effective means for the hemodynamic evaluation of small aortic prosthetic valves [8-15]. This pharmacological test does not have the above limitations. This study was carried out to evaluate rest and DSE hemodynamic response of 17 mm St. Jude Medical Regent (SJMR-17 mm) aortic prosthesis in relatively aged patients. The SJM Regent is a new-generation mechanical heart valve that represents the design evolution of the St. Jude Hemodynamic Plus (SJM HP) series. It is constructed of pyrolytic carbon which has a modified external profile that achieves a larger geometric orifice area without changing the existing design of the pivot mechanism or blood-contact surface area. The SJMR-17 mm valve, having a large actual (nominal) orifice area (AOA) as provided by manufacturer equivalent to a standard valve one size larger, seemed appropriate to be implanted and evaluated in relatively aged patients with aortic valve stenosis and small aortic root, where other alternatives, such as annulus enlargement, in order to make space for a larger valve prosthesis were not suitable because of the increased operative risk.

## Methods

### Patient population

The study population consisted of nineteen consecutive patients of mean age 69.2 ± 7.3 years (2 men, 17 women), who 36 ± 12 months before had received a SJMR-17 mm aortic valve, after sizing the aortic annulus and deciding not to attempt to enlarging it. This cohort represents **7% **of patients (19/265) who underwent aortic valve replacement (AVR) over four years at our Centre. AVR had been performed for rheumatic or degenerative valve disease resulting in severe stenosis. In one patient with degenerative mitral valve disease and moderate-severe valvular regurgitation concomitant valve replacement had been performed and in two patients with significant CAD single coronary artery by-pass grafting. Four patients with degenerative mitral valve disease and less than moderate mitral regurgitation had been treated with AVR alone. All patients but one were in sinus rhythm at the time of the study; none had had a myocardial infarction or angina pectoris after the operation. Demographic data are summarized in Table 1. All patients underwent basal echocardiography before cardiac surgery and 1 month after surgery and were controlled every year by clinical examinations. At mean distance of 24 months after surgery patients were enrolled in the study and underwent rest echocardiography and DSE. Beta-blockers were discontinued in all patients 24 hours before the test, whilst patients on ace-inhibitors and calcium antagonists continued their medication. Informed written consent was obtained from all patients.

**Table 1:** Demographic and Clinical variables.

Variables	N° (%)
Females/males	17/2
Mean age (years)	69.2 ± 7.3
Mean Body Surface Area (m2)	1.68 ± 0.2
Mean Body Mass Index (Kg/m2)	24.6 ± 7.5
Diabetes	4 (21)
Coronary Artery Disease history	4 (21)
Hypertension	8 (42)
Hypothyroidism	2 (10.5)
Smoking history	5 (26.3)
Previous Transient Ischemic Attack	4 (21)
Previous carotid endarterectomy	1 (5.2)
Peripheral vascular disease	2 (10.5)
Atrial fibrillation	1 (5.2)
Chronic obstructive pulmonary disease	4 (21)
Mean New York Heart Association Functional class	2.75 ± 0.86
Mean Canadian cardiovascular Class	1.63 ± 0.72
Left ventricular ejection fraction <35%	1 (5.2)
Mitral valve disease	5 (26.3)

### Study protocol

All studies (pre-operative, post-operative and follow-up) were performed with the use of 2.5–3.5 MHz transducer interfaced to the SONOS 5500 (Agilent Technologies, Andover, Mass) by same physicians (G.M., C.M., G.P.). The baseline study with standard M-Mode and 2-D measurements was completed according ASE criteria [16] and left ventricular mass (LVM) as well as left ventricular mass index (LVMi) were measured. Dobutamine was then infused intravenously starting at 5 microg/Kg/min and increased by 5 microg/Kg/min at 5 min intervals up to 40 microg/Kg/min. The DSE was terminated if any of the following end-points were met: (1) target heart rate >85% of maximal predicted, (2) angina or progressive dyspnoea, (3) 2-mm ST-segment depression 80 msec after the J point, (4) hypertension (systolic blood pressure >220, diastolic blood pressure >120 mmHg), (5) hypotension (drop in systolic blood pressure >30 mmHg), (6) frequent or polymorphous ventricular ectopic beats, (7) supraventricular tachyarrhythmias. Heart rate (HR) and a 12-lead ECG were recorded continuously; blood pressure, ejection fraction (EF), cardiac output (CO), peak and mean pressure gradients (MxPG, MnPG), effective orifice area (EOA), effective orifice area index (EOAi) as well as Doppler velocity index (DVI) were measured at baseline and at the end of each increment of dobutamine.

### Measurements and calculations

The MxPG across the prosthesis was estimated by the modified Bernoulli equation (4 V^2^); the MnPG was derived by planimetry of the Doppler envelope. Measurements from at least three velocity envelopes were averaged to assure consistency. The EOA was calculated by the continuity equation: EOA (cm^2^)= CSA LVOT × V LVOT integral/V Transprosthetic integral, where CSA is cross-sectional area, LVOT is left ventricular outflow tract, and V is velocity integral. Assuming a circular shape, the CSA LVOT was calculated as: 3.14 × (D/2)^2^, where D is the inner diameter of the left ventricular outflow tract. The V LVOT was obtained with pulsed-wave Doppler in the left ventricular outflow tract proximal to the aortic prosthesis from the apical five-chamber view; the V Transprosthetic was obtained with continuous-wave Doppler from the apical five-chamber view. The EOAi was calculated by the formula EOA/BSA (body surface area). LVM was derived from Devereux's formula and LVMi from LVM/BSA. The DVI was calculated by the formula: V integral LVOT/V integral transprosthetic (normal value >0.40). The CO was derived from the formula: stroke volume (CSA × V LVOT integral) X HR.

### Statistical analysis

Group statistics were expressed as mean ± SD. Paired Student's test was used to measure the variations of the parameters documented between baseline and follow-up. The non-linear relation between mean trans-prosthetic gradients at rest and EOAi was analysed by means of scatterplots and exponential curve estimation regression statistics. All analyses were performed by using SPSS 11.0 statistical software. A p value < 0.05 was considered significant.

## Results

**Figure 1:**
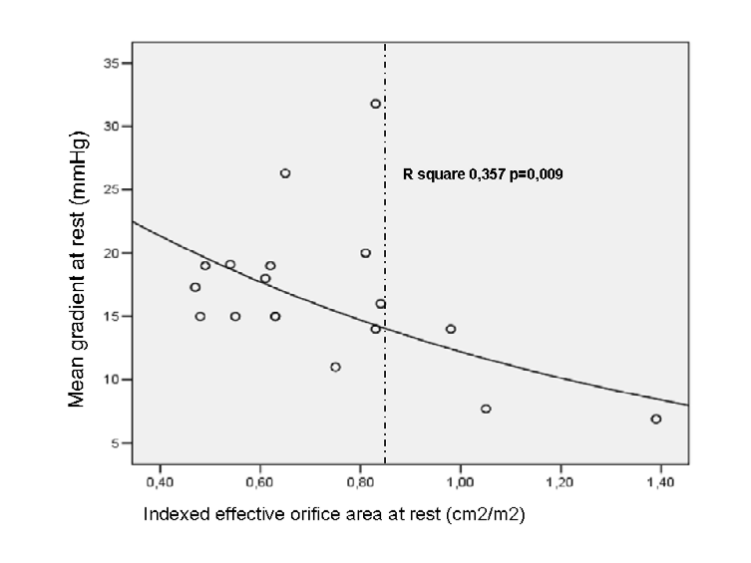
Relation between mean transprosthetic gradient at rest and indexed effective orifice area.

**Figure 2:**
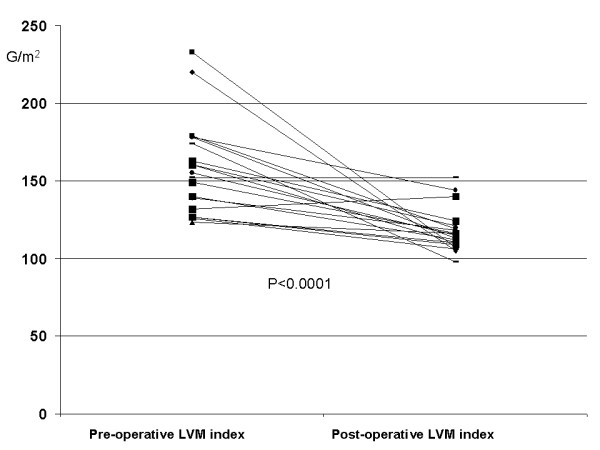
Pre- and post-operative left ventricular mass index values in individual patients (LVMi pre and post).

Pre-operative and post-operative echocardiographic data are reported in Table 2. At the time of the study the mean NYHA class was significantly lower than pre-operatively (1.3 ± 0.6 vs 2.75 ± 0.86, p < 0.001). The basal transprosthetic mean and maximal flow velocities were, respectively, 1.7 ± 0.2 and 2.6 ± 0.3 m/sec; consequently MnPG and peak MxPG were 16.6 ± 5.8 and 29.2 ± 7.1 mmHg; EOA and EOAi resulted 1.14 ± 0.3 cm^2 ^and 0.76 ± 0.2 cm^2^/m^2^; DVI was normal (0.50 ± 0.1) (Table 3). A non-linear relation between mean transprosthetic gradient at rest and EOAi was found, being patients with EOAi <0.85 cm^2^/m^2 ^on the steep portion of the curve, where gradients are relatively high (Figure 1). LVM and LVMi decreased from pre-operative values of 258 ± 43g and 157.4 ± 27.7g/m^2^ to 191 ± 23.8g and 114.5 ± 10.6 g/m^2^, respectively (p < 0.00001 and <0.0001) (Figure 2). Individual data regarding a possible link between rest and stress gradients, EOAi and regression of hypertrophy are reported in Table 4. All patients completed DSE without complications. Four patients had occasional premature atrial and/or ventricular beats, which did not preclude them from completing the test. With dobutamine, HR increased from a baseline of 64.5 ± 10 to 100.6 ± 28 beats/min (p < 0.001); CO increased from a baseline of 4.7 ± 1.6 L/min to 8.2 ± 2 L/min (p < 0.0001); EF increased from a baseline of 58.4 ± 8% to 68 ± 9.9% (p < 0.008). Basal systolic and diastolic blood pressure did not change significantly during test, whilst the double product increased significantly. At peak dobutamine, the mean and maximal flow velocities increased from baseline to 2.9 ± 0.4 and 4.5 ± 0.5 m/sec., respectively (p < 0.0001 and <0.0001); the MnPG and MxPG increased significantly from baseline, achieving, respectively, 43.2 ± 12.7 and 83.4 ± 21.9 mmHg (p < 0.0001 and p < 0.0001). EOA, EOAi and DVI changed during the test. At peak dobutamine, these parameters had an insignificant increase, EOA and EOAi from baseline to stress 1.2 ± 0.4 cm^2 ^and 0.75 ± 0.3 cm^2^/m^2 ^(p = 0.34 and 0.45) respectively, the DVI from baseline to stress 0.48 ± 0.1 (p = 0.72). Changes of all parameters are summarized in Table 3. Two out of the 19 patients developed significant subvalvular or intraventricular gradients during DSE. The mean NYHA class did not change significantly during the test or soon after.

**Table 2:** Pre- and post-operative echocardiographic data.

Variables	before surgery	after surgery	p-value
BSA m2	1.6 ± 0.2		
LVOT mm	17.5 ± 1.5		
Effective Orifice Area (cm2)	0.6 ± 0.2	1.1 ± 0.3	<0.0001
Indexed Effective Orifice Area (cm2/m2)	0.36 ± 0.9	0.7 ± 0.2	<0.0001
Maximal velocity (m/sec)	4.6 ± 0.6	2.7 ± 0.4	<0.0001
Mean velocity (m/sec)	3.6 ± 0.5	1.8 ± 0.3	<0.0001
Maximal gradient (mmHg)	88.2 ± 20.3	32.6 ± 11.3	<0.0001
Mean gradient (mmHg)	53.5 ± 13.6	16.6 ± 5.8	<0.0001
VTI LVOT (cm)	25.5 ± 6.9	27.45 ± 9.6	NS
VTI transvalvular (cm)	105.3 ± 20.1	57.2 ± 15.79	<0.0001
Doppler Velocity Index	0.24 ± 0.1	0.47 ± 0.1	<0.0001
Left Ventricular Mass (g)	258 ± 43	NA	
Indexed Left Ventricular Mass (g/m2)	157.4 ± 27.7	NA	

**Table 3:** Echocardiographic data at rest and under DSE at follow up.

Variables	at rest	under peak stress	p-value
Effective Orifice Area (cm2)	1.14 ± 0.3	1.2 ± 0.4	NS
Indexed Effective Orifice Area (cm2/m2)	0.76 ± 0.20	0.75 ± 0.3	NS
Maximal velocity (m/sec)	2.6 ± 0.3	4.5 ± 0.5	<0.0001
Mean velocity (m/sec)	1.7 ± 0.2	2.9 ± 0.4	<0.0001
Maximal gradient (mmHg)	29.2 ± 7.1	83.4 ± 21.9	<0.0001
Mean gradient (mmHg)	16.6 ± 5.8	43.2 ± 12.7	<0.0001
Doppler Velocity Index	0.50 ± 0.1	0.48 ± 0.1	NS
VTI LVOT (cm)	32.1 ± 10.5	34.2 ± 7.7	NS
VTI transprosthetic (cm)	62.8 ± 13.7	72.5 ± 17.3	0.032
Left Ventricular Ejection Fraction	58.4 ± 8	68 ± 9.9	<0.008
Cardiac Output (L/min)	4.7 ± 1.6	8.2 ± 2	<0.0001
Heart rate (b/min)	64.5 ± 10	100.6 ± 28	<0.001
Systolic pressure (mmHg)	139 ± 23	152 ± 26	NS
Diastolic pressure (mmHg)	77 ± 10.6	77 ± 12.5	NS
Double Product	8978 ± 2184	15225 ± 5147	<0.0001
Left Ventricular Mass (g)	191 ± 23.8	NA	
Indexed Left Ventricular Mass (g/m2)	114 ± 10.6	NA	

**Table 4:** Rest and peak gradients, indexed effective orifice area and regression of left ventricular hypertrophy in individual patients.

Patient	Rest Max PG (mmHg)	Rest Mean PG (mmHg)	Stress Max PG (mmHg)	Stress Mean PG (mmHg)	Rest Indexed EOA (cm2/m2)	Delta LVMi (g/m2)
1	29,00	15,00	50,00	23,00	0,63	-115
2	16,00	6,90	51,00	22,50	1,39	-126
3	35,50	17,30	50,00	28,00	0,47	-7
4	39,40	19,10	48,00	24,00	0,54	-33
5	62,70	31,80	65,00	30,00	0,83	-50
6	14,00	7,70	40,00	21,00	1,05	-40
7	44,10	26,30	58,00	28,00	0,65	8
8	29,00	15,00	45,00	22,00	0,63	-65
9	38,00	18,00	45,00	30,00	0,61	0
10	29,00	14,00	113,00	58,00	0,98	-17
11	35,00	19,00	83,00	44,00	0,49	-60
12	35,00	19,00	72,00	32,00	0,62	-39
13	30,00	15,00	114,00	58,00	0,55	-18
14	42,00	20,00	93,00	39,00	0,81	-39
15	28,00	16,00	65,00	38,00	0,84	-34
16	23,00	11,00	101,00	59,00	0,75	-28
17	25,00	14,00	62,00	33,00	0,83	-20
18	29,00	15,00	73,00	41,00	0,48	-76
19	35,00	17,00	63,00	28,00	0,71	-21

EOA: Effective orifice area; PG: pressure gradient; LVMi: left ventricular mass index

## Discussion

Two-dimensional and Doppler echocardiography is an accurate, reliable and non invasive tool for assessing prosthetic heart valves [1,2]. However, the ideal means for testing valve function requires rest and stress hemodynamic evaluation, under various flow conditions [2,6]. Recently, DSE has been proposed as an alternative means for evaluating valve hemodynamics [8-15]. In this study we used basal and dobutamine 2-dimensional and Doppler echocardiography for assessing aortic SJMR-17 mm prostheses, implanted in patients with a small aortic root, the use of which is controversial because of the small orifice area. In our experience SJMR-17 mm prostheses showed rest mean and peak gradients of 16.6 ± 5.8 and 29.2 ± 7.1 mmHg similar to the gradients reported in literature for SJM standard or HP valves of larger sizes (19–21 mm), respectively of 17.2 ± 3.3 and 33 ± 4 mmHg [4,9,11,12,15,18,19], and slightly higher when compared to other larger (21–23 mm) bileaflet mechanical valves, respectively of 15 ± 2.6 and 27.7 ± 4 mmHg [4,5,9,12-15], although there was a large overlap between the values of these variables. When we compared EOA and EOAi of SJMR-17 mm with the reported results of larger (19–23 mm) mechanical valves, we found values slightly lower respectively 1.14 ± 0.3 cm^2 ^vs 1.21 ± 0.19 cm^2 ^and 0.76 ± 0.2 cm^2^/m^2 ^vs 0.88 ± 0.2 cm^2^/m^2^. The finding of average EOAi < 0.85 cm^2^/m^2 ^might suggest that the majority of patients had a mild prosthesis-patient mismatch (PPM) [17]. During DSE mean and peak flow velocity, and mean and peak gradients rose markedly. Similar results were obtained in previous studies which evaluated prosthetic valves of different types and sizes under stress conditions, induced by exercise or dobutamine echocardiography [4,9,11-15]. These studies clearly demonstrated a strong, inverse correlation between prosthetic size and basal and stress gradients, namely smaller valves with more elevated mean and peak gradients. Large BSA and young age have also been considered as predictors of unfavorable hemodynamics [5,11,12,17]. The relation between BSA and gradients is still controversial [5,11,12]. No correlation was found in our study. Young age (<50 years), especially in patients who are physically active, has been reported as predictor of potential PPM [11,12]. It has therefore been suggested [11,13,14,20] that in patients requiring a 19 mm valve or smaller, prostheses with the largest actual (nominal) orifice area (AOA) as provided by manufacturer, or other types of valve prostheses, i.e. stentless porcine [4,13,14,21-25], aortic homograft [24] or pulmonary autograft [22,23] should be considered. As an alternative patients could be subjected to an aortic root enlargement procedure [25]. However, the increased operative risk of this procedure must be considered. In our study BSA and age of patients were 1.68 ± 0.2 m^2 ^and 69.2 ± 7.3 years, respectively and SJMR-17 mm prostheses, having an AOA equivalent to a standard valve one size larger, seemed appropriate. Our patients are older than those usually enrolled in published studies, in which the mean age is 62.5 ± 5.5 years [4,5,8-13,15]. The transprosthetic valve gradient depends on the valve type and size, the diastolic filling period, and the ventricular loading conditions. Therefore, it should be noted that high gradients do not necessarily mean prosthetic stenosis. Since the volumetric flow in the outflow tract equals the volumetric flow through the prosthetic valve (Q LVOT=Q prosthesis), the rise in cardiac output generated by DSE increases the flow velocities on the two sides of the valve orifice, maintaining the EOA basically unchanged [9,26]. This principle of conservation of mass was clearly demonstrated in our study when the continuity equation (Q LVOT × A LVOT= Q transprosthetic × A transprosthetic) was applied at baseline and at peak stress: the aortic valve area remained relatively unchanged despite a significant increase in transprosthetic gradients. Such a finding is highly expected as mechanical valves, because of their stiff components, are less prone to accommodating larger stroke volume by increasing their effective area. These results confirm previous data derived from patients with larger size mechanical aortic prosthesis [4,9,11-15], suggesting that a mild-moderate PPM could not always be an issue of clinical relevance[17]. Some authors have found that PPM leads to higher mortality rates[17], others have found no effects on overall mortality[27,28], but its clinical impact probably seems to be related to both severity of LV workload and function, thus suggesting the fact that a diseased ventricle is much more sensitive to increased afterload[17]. The DVI, which is a further guide to valve orifice behaviour, independent of measurement of LVOT diameter [29], has been evaluated only in few studies [15,30]. In our study it remained substantially unchanged during stress, according to EOA and EOAi. During dobutamine infusion CO, EF, HR and double product increased significantly, confirming published data [4,9,12,13,15].

The influence of prosthesis size on change in LVM remains controversial [5,31-33]. Some studies found a strong correlation between prosthetic size and left ventricular hypertrophy (LVH) regression [5,17,18,31], others did not support this finding [32,33]. Moreover, other studies found that the degree of mass regression may vary markedly from one patient to another[17]. In this series the LVM and LVMi decreased significantly from pre-operative, returning to a normal range. Thus the SJMR-17 mm prosthesis, which increases EOA of the aortic valve and consequently reduces the pressure gradient, probably eliminates one of the factors stimulating LVH. These findings seem to confirm the positive effect of AVR on LVH irrespective of prosthesis size and of mild PPM. Furthermore, the increase in maximal and mean trans-prosthetic gradients during DSE, which mimics stress conditions of patient's daily activities, seems to be without clinical importance in this sample population, represented however by relatively aged patients. Two patients who had isolated AVR developed significant dynamic outflow obstruction during DSE, without clinical symptoms. This phenomenon has been observed in patients with proven or suspected CAD [34-36] (prevalence of 3.8–7.5%) or in patients who have undergone concomitant mitral valve repair [37]. The suspected mechanisms are: increased myocardial contractility, decreased venous return to the left ventricle, mitral valve systolic anterior motion or peculiar characteristics of left ventricular geometry. This finding was detected in up to 60% of patients with a small aortic prosthesis by Hunziker et al. [11], but has not been confirmed by other studies [4,9,12-14]. To our knowledge, this is the first report dealing with patients with 17 mm prosthetic aortic valves. The use of dobutamine as in our protocol was not associated with side effects, confirming previous data [9]. It would appear that this test can be safely used in relatively aged patients, which represent a growing population requiring aortic valve replacement. An additional advantage of using the "full" protocol is the ability to assess coronary artery disease, by analysis of left ventricle segmental wall motion under stress conditions.

## Limitations of the study

The small number of patients examined demonstrates the rarity of patients potentially requiring a 17 mm aortic valve prosthesis. In this experience the 17 mm aortic valve prosthesis was implanted in relatively aged patients, older than those usually enrolled in published studies. It is therefore difficult to differentiate the hemodynamic response in younger patients who have a more active life. Methodologically, a major limitation of continuous-wave Doppler is the possibility of overestimating valvular flow velocity and pressure gradients and underestimating the valve area by continuity equation, as a consequence of pressure-recovery phenomenon. Since the rest and dobutamine prosthetic assessment are both subject to the same limitation, the ensuing intra-individual comparison that follows is statistically valid.

## Conclusion

In relatively aged patients with a small aortic root, the SJMR-17 mm valves can be implanted, because of their satisfactory hemodynamic profile at rest and under pharmacological stress conditions. Basal and dobutamine echocardiography is a useful and effective means for evaluating prosthesis hemodynamics (gradients, EOA, DVI and indexed values) and for monitoring the expected LVH regression. A standard methodology for studying aortic prostheses in basal conditions, using these echocardiographic paramethers, is warranted.

## Competing interests

The author(s) declare that they have no competing interests.

## Authors' contributions

GM and FM made substantial contributions to conception and design of the study, analysis and interpretation of data; GM, CM, VC, DM, GP, and GC made substantial contributions in acquisition of clinical, surgical and echocardiographic data; GM and GP performed the statistical analysis; GM, GP and FM have been involved in drafting the manuscript and revising it critically ; FM has given final approval of the version to be published.
